# Identification of influenza polymerase inhibitors targeting C-terminal domain of PA through surface plasmon resonance screening

**DOI:** 10.1038/s41598-018-20772-9

**Published:** 2018-02-02

**Authors:** Chun-Yeung Lo, Olive Tin-Wai Li, Wen-Ping Tang, Chun Hu, Guo Xin Wang, Jacky Chi-Ki Ngo, David Chi-Cheong Wan, Leo Lit-Man Poon, Pang-Chui Shaw

**Affiliations:** 1 Centre for Protein Science and Crystallography, School of Life Sciences, The Chinese University of Hong Kong, Shatin, Hong Kong SAR China; 2 Centre of Influenza Research, School of Public Health, Li Ka Shing Faculty of Medicine, The University of Hong Kong, Shatin, Hong Kong SAR China; 3 The School of Pharmaceutical Engineering, Shenyang Pharmaceutical University, Shenyang, China; 4 Research Center of Plasmonic and Near-Infrared Science, Research Institute of Tsinghua University in Shenzhen, Shenzhen, China; 5 School of Biomedical Sciences, The Chinese University of Hong Kong, Shatin, Hong Kong SAR China

## Abstract

Currently, many strains of influenza A virus have developed resistance against anti-influenza drugs, and it is essential to find new chemicals to combat this virus. The influenza polymerase with three proteins, PA, PB1 and PB2, is a crucial component of the viral ribonucleoprotein (RNP) complex. Here, we report the identification of a hit compound **221** by surface plasmon resonance (SPR) direct binding screening on the C-terminal of PA (PAC). Compound 221 can subdue influenza RNP activities and attenuate influenza virus replication. Its analogs were subsequently investigated and twelve of them could attenuate RNP activities. One of the analogs, compound 312, impeded influenza A virus replication in Madin-Darby canine kidney cells with IC_50_ of 27.0 ± 16.8 μM. *In vitro* interaction assays showed that compound 312 bound directly to PAC with Kd of about 40 μM. Overall, the identification of novel PAC-targeting compounds provides new ground for drug design against influenza virus in the future.

## Introduction

Influenza virus is a major threat to the health of the community. The annual deaths caused by seasonal influenza epidemics range from 250000 to 500000^1^. The ever-changing nature of influenza virus by antigenic variations has been challenging for the development of an effective influenza drug. Oseltamivir-resistant strains were identified from the outbreaks of 2009 H1N1 pandemic^2^ and 2013 H7N9^3^. Recently, many human cases of avian influenza A H7N9 virus were confirmed in China^4^. Also, a highly pathogenic avian influenza A H5N8 virus has also been rapidly spreading since June 2016^5^. All these call for attention to the urgency of developing new antivirals.

The influenza RNA-dependent RNA polymerase (RdRP), consisting PA, PB1 and PB2 subunits, is a component of the viral ribonucleoprotein (RNP) complex that is crucial for viral transcription and replication^6^. Nucleic acid polymerase activity resides in the PB1 subunit, which forms the core of the complex^7,8^. Both PA and PB2 are involved in various accessory functions that are essential for the transcription and replication of the viral genome. The highly conserved nature of RdRP makes it an attractive target for drug discovery^9^.

PA, one of the RdRP subunits, consists of two domains-the N-terminal endonuclease domain (residues 1–195) and the C-terminal domain (PAC) (residues 257–716). They are connected by an extensive linker that wraps around PB1 complex^7,8^. PAC contains a hydrophobic groove that interacts with the N-terminus of PB1 subunit. This PB1-binding groove is highly conserved among different viral strains^10,11^. PA-PB1 interaction is necessary for the assembly of the RdRP complex, and it is also involved in the nuclear import of both proteins^12^. Several studies have demonstrated that the disruption of PA-PB1 interaction could impede influenza virus replication^13–20^. Furthermore, PAC has been reported to have protease activity^21^. Mutational study has also revealed that several residues in PAC are important for the transcription and replication activity of influenza ribonucleoprotein (RNP)^22^. All these suggest that PAC could be a feasible target for drug screening.

In the present study, we have conducted a surface plasmon resonance (SPR) screening of an in-house library to identify hit compounds targeting PAC. SPR is a biophysical method for characterizing label-free macromolecular interaction. It is highly sensitive and could provide quantitative analysis of the interactions between protein and small molecules. Direct binding screening using SPR has been employed on various protein targets, using libraries of several hundred compounds to several thousand^23–27^. From our screening study, two hit compounds (compound **221** and **283**) were discovered to attenuate RNP activities and inhibit influenza virus. Analogs of compound **221** were further evaluated and one of them (compound **312**) was characterized as a promising inhibitor of influenza virus.

## Results

### SPR screening for compounds binding to PAC *in vitro*

**Figure 1:**
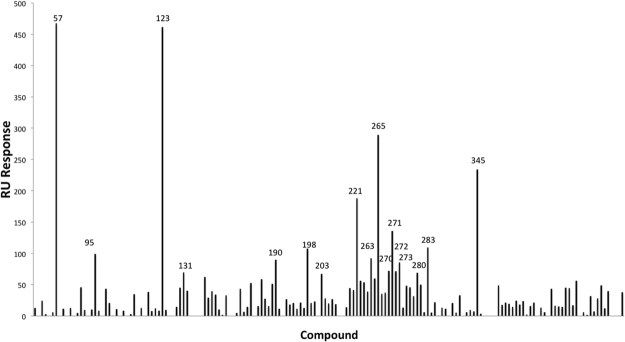
SPR screening for PAC. 165 compounds were screened against PAC by SPR using Biacore3000. 17 compounds with the highest RU responses were chosen for further examination.

One hundred and sixty-five compounds from our in-house library were screened against PAC by SPR using Biacore 3000. The RU responses of test compounds after DMSO calibration were recorded and ranked (Fig. 1). The top 10% (17 compounds) were selected for further investigation. Their structures are summarized in Supplementary Table 1.

### Cytotoxicity of compounds

Cytotoxicity of selected compounds were evaluated in both 293T and MDCK cells. Most of them have CC50 over 100 μM. The cytotoxicity of the hit compounds is summarized in Supplementary Table 2.

### Identification of hits that inhibit ribonucleoprotein (RNP) function and impede influenza virus replication

**Figure 2:**
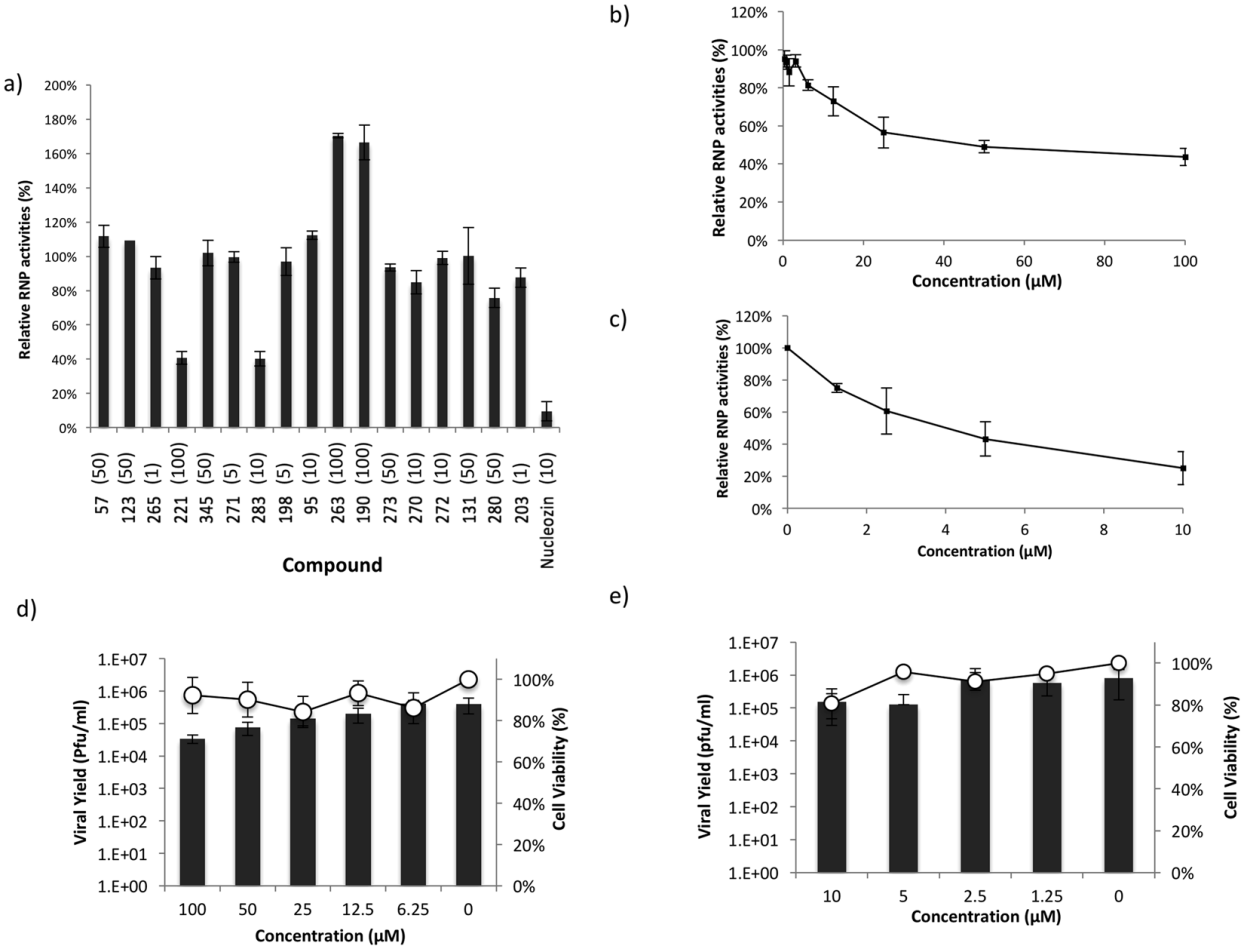
Evaluation of hit compounds. (**a**) 17 hit compounds obtained from SPR screening was evaluated by RNP assays. The highest non-cytotoxic concentration (in μM) of each compound is shown in bracket. Compounds **221** and **283** showed significant attenuation of RNP. Nucleozin was included as a positive control. (**b**) Compound **221** and (**c**) compound **283** elicited dose dependent inhibition of influenza RNP activity. (**d**) Compound **221** and (**e**) compound **283** showed dose dependent inhibition of influenza A/WSN/33 at MOI 0.01. Bar chart represents viral yield while XY curve represents cell viability. Results were obtained from three independent experiments.

Next we employed an RNP reconstitution reporter assay to investigate if the hit compounds can reduce RNP transcriptional activities. The highest non-cytotoxic concentration of each compound was used in the assay. Among the 17 hit compounds, compound **221** and **283** caused a significant attenuation of RNP transcriptional activities (Fig. 2a). Both compounds exhibited dose dependent inhibition of RNP activity (Fig. 2b,c), with compound **221** having IC_50_ of 68.8 ± 1.9 μM and compound **283** having IC_50_ of 8.80 ± 2.4 μM. Furthermore, compound **221** and **283** could impede influenza virus (A/WSN/33) replication in viral yield reduction assay, with IC_50_ 30.58 ± 21.37 and 1.67 ± 1.27 μM respectively (Fig. 2d,e).

### Biological activity of compound 221 analogs

According to the chemical structure of compound **221**, 10 analogs were designed and synthesized. Another 13 commercially available analogs were also purchased. Their structures are summarized in Table 1.

**Table 1:** Chemical structures of 221 analogs.

	Compound	R1	R2			R3
Synthetic compounds group 1	S1a		-H			-H
	S1b		-H			-CH_3_
	S1c		-CH_3_			-H
	S1d		-F			-H
**Synthetic compounds group 2**	**Compound**	**R1**	**R2**			**R3**
	**221**	-CH_3_				-H
	**S2a**	-CH_2_CH_2_OH				-H
	**S2b**	-H				-H
	**S2c**	-CH_3_				-H
	**S2d**	-CH_3_				-CH_3_
	**S2e**					-H
	**S2f**	-CH_3_				-H
**Commercially available analogs group 1**	**Compound**	**R1**	**R2**	**R3**	**R4**	**R5**
	**390**		-H	-H	-H	-CH_3_
	**391**		-H	-OCH_3_	-OH	
	**394**	-CH_2_CH_3_	-H	-H	-H	
	**395**		-CH_3_	-H	-H	
	**396**	-CH_2_CH_3_	-H	-F	-H	
	**397**		-H	-CH_3_	-H	-CH_3_
**Commercially available analogs group 2**	**Compound**	**R1**	**R2**	**R3**		**R4**
	221	-CH3	=O			-OH
	312	-H	=O			-OH
	385	-CH3	=O			-OH
	387	-H	=O			-OH
**Uncategorized commercially available analogs**	**Compound**	**Chemical** illucture				
	**310**					
	**384**					
	**389**					
	**392**					

**Figure 3:**
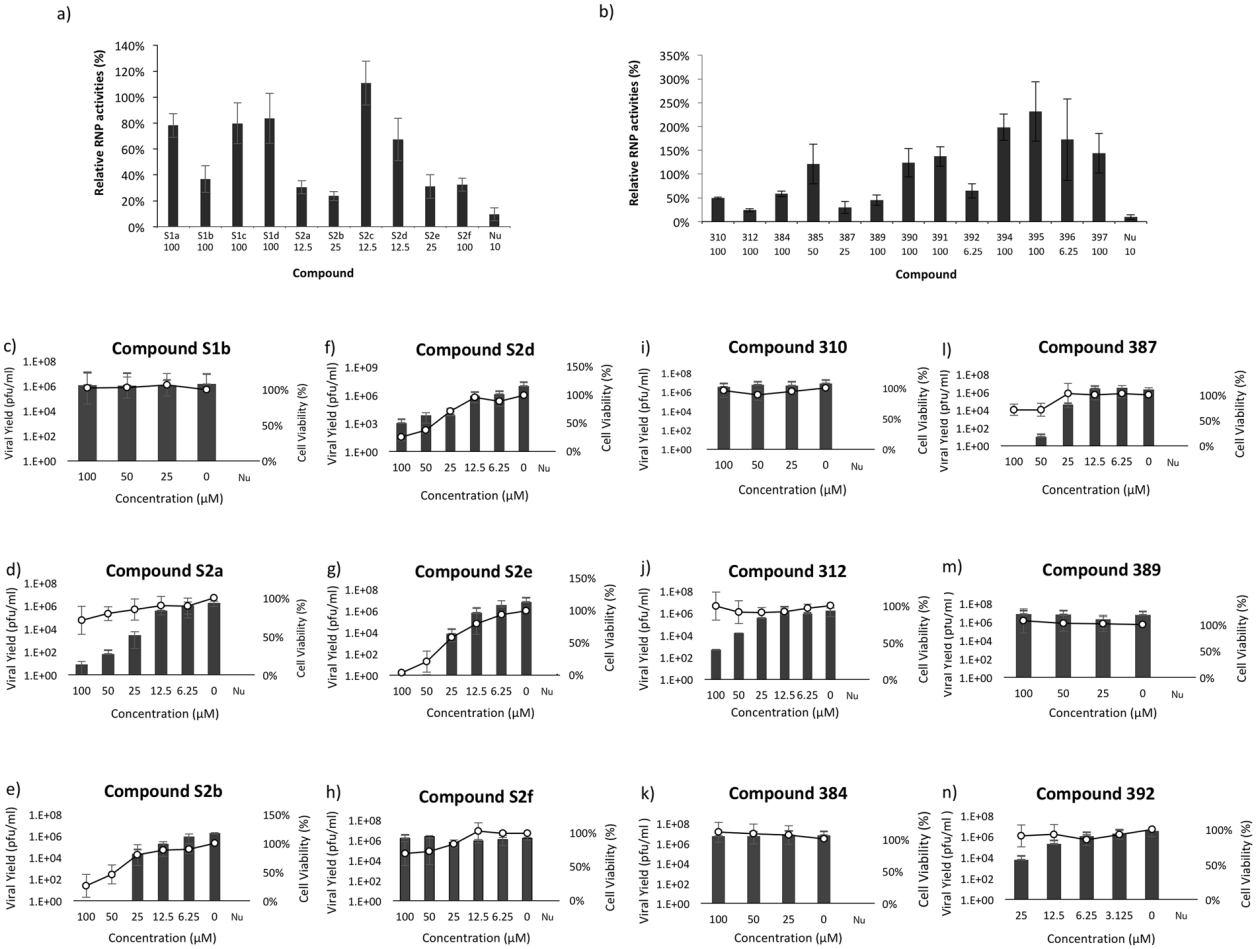
Evaluation of analogs of compound 221. (**a**) RNP screening of analogs of 221 obtained by chemical synthesis. Ten analogs of compound **221** were designed and synthesized. Six of them exhibited significant reduction of RNP activities. The highest non-cytotoxic concentration (in μM) of each compound is shown below its name. (**b**) Thirteen commercially available analogs of compound **221** were evaluated by RNP assays. Six of them exhibited significant reduction of RNP activities. The highest non-cytotoxic concentration of each compound is shown belowits name. Dose dependent viral yield reduction assays of (**c**) **S1b**, (**d**) **S2a**, (**e**) **S2b**, (**f**) **S2d**, (**g**) **S2e**, (**h**) **S2f**, (**i**) **310**, (**j**) **312**, (**k**) **384**, (**l**) **387**, (**m**) **389**, (**n**) **392**. Bar chart represents viral yield while XY curve represents cell viability. Nucleozin (Nu) at 25 μM was used as positive control. Results were obtained from three independent experiments.

RNP screening showed that 12 out of the 23 analogs were effective in attenuating influenza RNP activities (Fig. 3a,b). All effective compounds exhibited dose dependent inhibition of RNP activity, most of them having IC_50_ below 100 μM (Table 2).

**Table 2:** Properties of analogs of compound 221.

	Compound	RNP assay		Viral Yield Reduction Assay		MST	SPR
		IC_50_^a^ (μM)	CC_50_^b^ (μM)	IC_50_^c^ (μM)	CC_50_^d^ (μM)	Kd^e^ (μM)	Kd^f^ (μM)
	**221**	68.8 ± 1.9	>100	30.58 ± 21.37	>100	N.D.	N.D.
Analogs obtained through chemical modification	**S1b**	87.8 ± 21.8	>100	>100	>100	N.D.	N.D.
	**S2a**	9.69 ± 3.00	84.6 ± 17.30	7.74 ± 6.02	>100	1.77 ± 0.78	N.D.
	**S2b**	14.2 ± 3.6	>100	4.96 ± 3.84	42.8 ± 9.0	16.2 ± 8.3	N.D.
	**S2d**	>12.5	66.6 ± 6.09	3.18 ± 2.44	35.6 ± 9.24	>25	N.D.
	**S2e**	6.69 ± 7.49	>100	5.11 ± 4.22	53.1 ± 9.25	50.9 ± 25.5	N.D.
	**S2f**	62.4 ± 22.4	>100	>100	>100	N.D.	N.D.
Commercially available analogs	**310**	98.45 ± 3.6	>100	>100	>100	N.D.	N.D.
	**312**	35.37 ± 4.3	>100	27.0 ± 16.8	>100	38.2 ± 5.5	37.7 ± 4.6
	**384**	>100	>100	>100	>100	N.D.	N.D.
	**389**	96.54 ± 28.7	>100	>100	>100	N.D.	N.D.
	**387**	15.45 ± 2.8	>100	6.45 ± 5.63	>100	46.3 ± 27.4	N.D.
	**392**	>6.25	62.66 ± 18.7	4.78 ± 5.63	87.03 ± 7.22	6.03 ± 1.05	N.D.

^a^IC_50_ is the concentration of test compound which produces 50% inhibition of RNP activity compared with DMSO control; reported values represent means ± standard deviation of data from three independent experiments.

^b^293T cells were incubated with test compounds for 24 hrs; CC_50_ is the concentration of test compounds which produces 50% cytotoxicity as determined by MTT assays. Reported values represent means ± standard deviation of data from three independent experiments.

^c^IC_50_ is the concentration of test compound which produces 50% inhibition of viral yield compared with DMSO control; reported values represent means ± standard deviation of data from three independent experiments.

^d^MDCK cells were incubated with test compounds for 24 hrs; CC_50_ is the concentration of test compounds which produces 50% cytotoxicity as determined by MTT assays. Reported values represent means ± standard deviation of data from three independent experiments.

^e^Kd were measured by MST and calculated by NanoTemper MO.Control using ‘Kd model’. Reported values represent Kd ± confidence level calculated by the software.

^f^Kd were measured by SPR and calculated by Biacore BiaEvaluation v 4.1 using ‘1:1 Langmuir binding model’. Reported values represent mean ± standard deviation of data from three independent experiments.

Viral yield reduction study with A/WSN/33 (0.01 MOI) shows that compound **S2a, S2b, S2d, S2e, 312, 387** and **392** exhibited dose-dependent inhibition of influenza virus (Fig. 3c–n). Most of their IC_50_ fall within low micromolar range (Table 2). However, the dose dependent effect of compounds **S2b**, **S2d** and **S2e** might not be reliable due to their correlation with cytotoxicity (Fig. 3e–g). Compound **S2a** and **387** exhibited slight cytotoxicity at their highest tested concentration (Fig. 3d,l).

For compounds with anti-influenza effect, their interactions with PAC were further confirmed and measured by microscale thermophoresis (MST) assay. Except for compound **S2d**, the Kd of other compounds all lie below 100 μM (Table 2 and Supplementary Figure S1–S6). As compound **S2d** showed significant intrinsic fluorescence which could interfere with MST measurement, we were unable to measure its MST signal at a concentration higher than 25 μM.

As compound **312** exhibited good dose-dependent inhibition of A/WSN/33 (IC_50_: 27.0 ± 16.8 μM) without showing any cytotoxicity (i.e. non-cytotoxic at 100 μM) (Fig. 3j), it was selected for further investigation.

### Biological activity of compound 312

**Figure 4:**
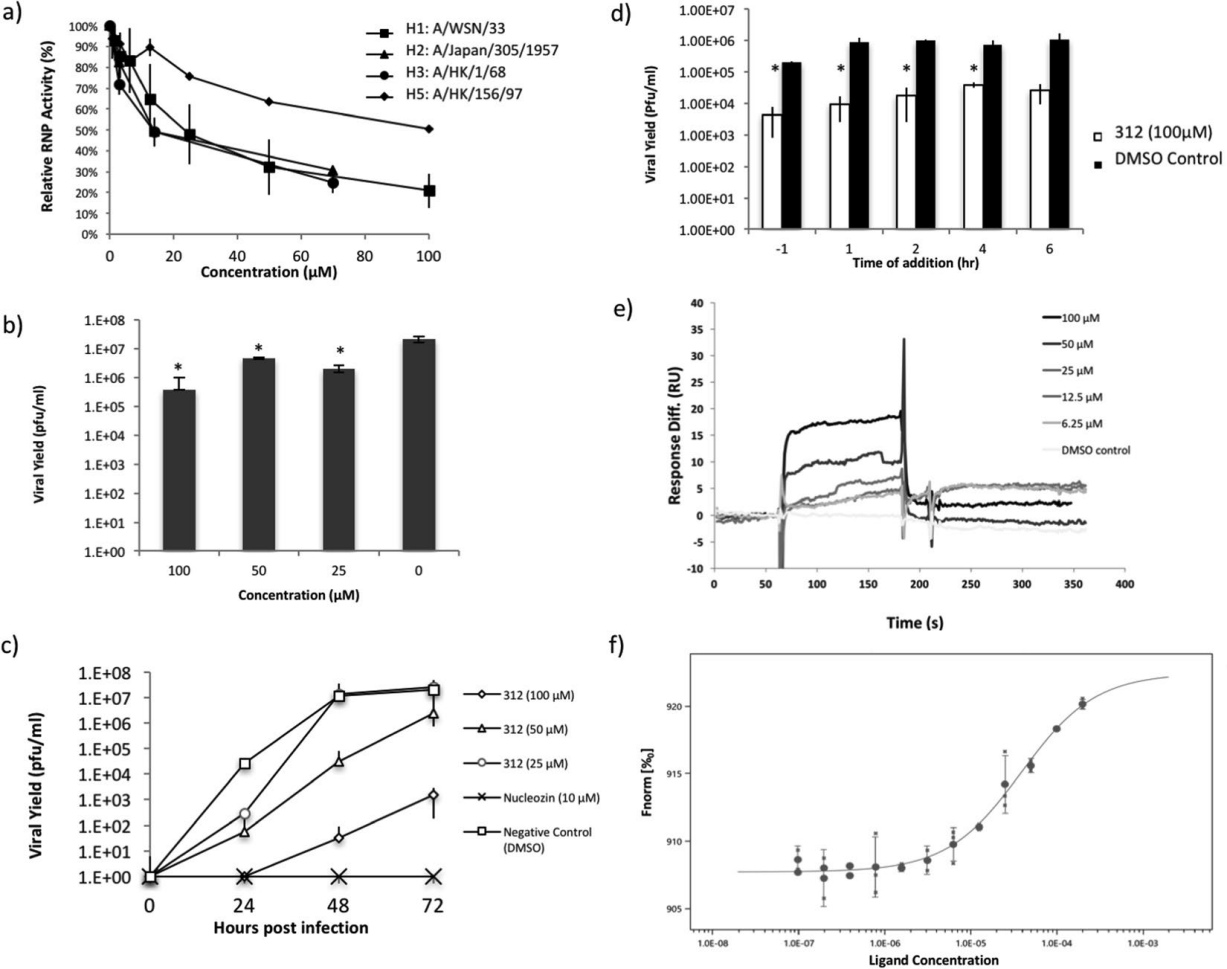
Evaluation of compound 312. (**a**) Compound **312** elicited dose dependent inhibition of RNP activities of various influenza subtypes. (**b**) Compound **312** showed inhibition of influenza A/PR/8/34. (**c**) Viral growth curve with compound **312**. Compound **312** could impede influenza A/WSN/33 propagation. (**d**) MDCK cells were infected with 0.01 MOI A/WSN/33 and incubated with 100 μM compound **312** at various time points. Compound **312** was able to attenuate virus when added post infection. (**e**) SPR study of compound **312** with PAC. Using the ‘1:1 Langmuir binding’ model by BIAevaluation v. 4.1 software, Kd value was estimated to be 37.7 ± 4.6 μM. Data shown represent the mean ± SD of three independent experiments. *Represents p < 0.05, **Represents p < 0.01. (**f**) MST study of compound 312 with PAC. Kd value was estimated to be 38.2 ± 5.5 μM by MO.Control software using Kd model.

RNP from various influenza strains (A/WSN/33 (H1N1), A/Japan/305/1957(H2N2), A/HK/1/68(H3N2) and A/HK/156/97(H5N1)) were used to evaluate compound **312**. Dose dependent inhibition of RNP activities is observed in all strains. The potencies of compound **312** on H1N1, H2N2 and H3N2 were similar, while it was significantly weaker on H5N1 RNP (Fig. 4a). Next, besides A/WSN/33, we also did viral yield assay with A/PR/8/34 virus. Compound **312** exhibited significant inhibition of A/PR/8/34 virus, reducing viral yield for about 57 fold at 100 μM (Fig. 4b).

To further confirm the effects of compound **312** on viral replication, we compared the viral growth curve in the presence or absence of compound **312**. MDCK cells were infected with 0.001 MOI H1N1 (A/WSN/33) virus and incubated with test compounds for 24, 48 and 72 hrs. The growth curves of influenza virus at 50 and 100 μM of compound **312** showed that it can significantly suppress viral growth (Fig. 4c). Suppression of influenza virus replication was also observed by the addition of compound **312** after viral infection (Fig. 4d).

The binding kinetics of compound **312** was further studied with SPR. Compound **312** elicited an increase of RU in a concentration dependent manner (Fig. 4e). Using the ‘1:1 Langmuir binding’ model by BIAevaluation v. 4.1 software, Kd value was estimated to be 37.7 ± 4.6 μM. The Kd measured by SPR is consistent with the Kd obtained from MST assay (38.2 ± 5.5 μM) (Fig. 4f).

## Discussion

In this study, we performed an SPR screening for PAC. Among the 165 compounds, we have identified two hits that can attenuate viral RNP activities. Both compounds showed dose dependent inhibition of RNP and viral yield with IC_50_ at micromolar level.

Compound 221 was selected for further investigation due to its structural simplicity and better solubility. Based on the structure of compound 221, ten analogs were designed and synthesized (Table 1). Compound S2a, S2b, S2c, S2d, S2e and S2f all have three aromatic groups attached to the core structure, while S1a, S1b, S1c and S1d have two of the aromatic groups removed.

All S1 compounds showed no cytotoxicity at 100 μM. However, all had lost their abilities to inhibit RNP activities except for S1b. Although all four of them retain a central two-ring core attached with an aromatic side group, their structural changes are more drastic than other synthetic compounds. Two aromatic rings were removed and a side group was added to position R1 (Table 1), which was not present in the structure of compound 221. These significant changes might explain the loss of activity.

On the other hand, in the class of S2 compounds, **S2a**, **S2b**, **S2c**, **S2d** and **S2e** highly resemble the original compound **221** (Table 1). Except for **S2c**, all had marked improvement in their potencies against RNP. However, they also exhibited increased cytotoxicity. Compounds **S2b, S2d** and **S2e** had CC_50_ at low micromolar level in MDCK cells. In contrast, compound **S2a** showed only slight increase in cytotoxicity at 100 μM. By inspecting the structures of these analogs, it can be shown **S2a** resembles compound **221** at position R2, having a phenyl side group without a methyl group attaching to it. In contrast, compounds **S2b, S2d** and **S2e** all have an extra methyl group. This might cause the discrepancy in cytotoxicity between these analogs. As for compound **S2f**, the side chain at position R2 has a carbon atom replaced by a nitrogen atom. This structural change might also explain its inability to attenuate influenza virus.

Next, 13 commercially available analogs of compound **221** were found in the Pubchem database using Tanimoto similarity index. Based on their core structures, these analogs can mostly be categorized into two groups (Table 1). Group 1 includes compounds **390**, **391**, **394**, **395**, **396** and **397**. Group 2 includes compounds **312**, **385** and **387**. The remaining compounds **310**, **384**, **389** and **392** cannot be fitted into both groups.

All the analogs in group 1 do not inhibit RNP activity. Its core structure has charges in it while the core structure of compound **221** is non-polar. This could explain why even some of these analogs highly resemble compound **221** (i.e. compounds **391** and **395**), yet they did not inhibit RNP.

For the uncategorized compounds (**310**, **384**, **389** and **392)**, all four were able to inhibit RNP activities. Compound **384** has one aromatic ring removed, and the original two-ring core structure has a five-member ring fused to it. Although having one side group removed, it still retained the capability to inhibit RNP, albeit to a less extent. The case is similar with compound **310** and compound **389**. Both have a two-ring core structure with only two aromatic side groups. Compound **310** and **389** inhibit RNP activities with IC_50_ of 98.45 ± 3.6 and 96.54 ± 28.7 μM respectively, both being less potent than compound **221**. In light of this, it could be conjectured that the aromatic group which the three (**310**, **384** and **389**) lack is non-essential but beneficial for their inhibitory effects. Furthermore, all three compounds were unable to inhibit influenza viral yield at 100 μM. Their inabilities to inhibit viral replication are consistent with their mild inhibition on RNP activities. The last uncategorized compound, **392**, has a tri-ring core attached with two aromatic side groups. It is more potent than compound **221**, inhibiting influenza virus with IC_50_ of 4.78 ± 5.63 μM, but exhibiting higher cytotoxicity in both 293T and MDCK cells.

In group 2, the core structure is identical to that of compound **221**. Among the three analogs, two of them (Compounds **312** and **387**) attenuate RNP activities. Compounds **312** and **387** both highly resemble compound **221**. Compound **387** differs from **221** by the removal of a methyl group from its core structure, while compound **312** replaces a benzyl group with a phenyl group. Both retain three aromatic side groups. Compound **312** and compound **387** inhibited RNP activities with IC_50_ of 35.37 ± 4.3 and 15.45 ± 2.8 μM respectively. Compound **387** is slightly cytotoxic in MDCK cells at above 50 μM (Fig. 3l). The failure of compound **385** to inhibit RNP activities could be due to its drastically different group at position R3.

Although it is evident that compound **221** and **312** can act on RNP and bind to PAC, their detail mechanism of action remains obscure. PAC has been shown to interact with: (1) PB1N-terminal^10,11^, (2) PB1 and PB2 at other positions^7,8^, (3) 5′ promoter region of viral RNA^7,8^, (4) small viral RNA (svRNA) and promote genome replication in a segment-specific manner^28^, (5) C-terminal domain of RNA polymerase II, which allows the RNP to recruit 5′-capped primers from nascent Pol II transcripts for viral genome transcription^29^.

**Figure 5:**
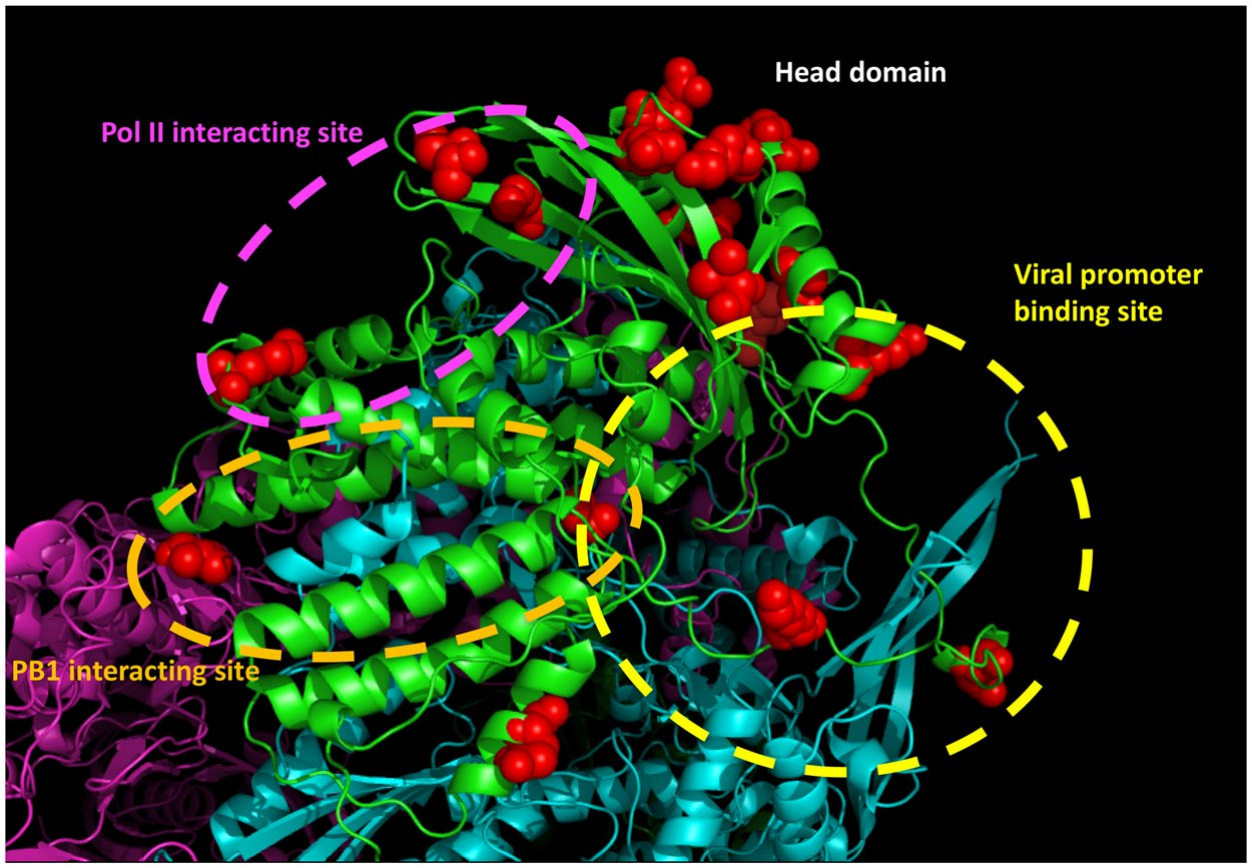
Location of the distinct residues of H5N1 PAC. Cartoon representation of bat influenza A polymerase complex structure (PDB: 4WSB). PA, PB1 and PB2 are colored in green, cyan and pink respectively. Seventeen residues where H5N1 PAC is distinct from H1N1, H2N2 and H3N2 PAC are shown as red spheres. The Pol II binding site on PAC is circled in pink, the viral promoter binding site is circled in yellow and the PB1 interacting site is circled in orange.

The discrepancy between the inhibition of H5N1 RNP activities and that of other influenza strains might shed some lights on the mechanism of action by compound **312**. Sequence alignment of A/WSN/33 (H1N1), A/Japan/305/1957 (H2N2), A/HK/1/68 (H3N2) and A/HK/156/97 (H5N1) PAC reveals that there are 17 residues where H5N1 PAC are distinct from the other three (Fig. 5 & Supplementary Fig. S7). The three-dimensional structure of bat influenza A RdRP complex (PDB: 4WSB) was inspected to understand the distribution and location of these residues^7^. It should be noted that residues 349–353 of H1N1, H2N2, H3N2 and H5N1 PA are not present in the H17N10 bat influenza polymerase, and the amino acid numbering of the later is different from the formers. Among the 17 residues, R497, T552 and R615 are in close proximity to the Pol II interacting residues (Residues 289, 454, 636 and 638; the interacting site is circled in pink); M367, E382, H394 and A651 are located at the viral promoter and svRNA binding region (circled in yellow); I602 and A651 reside in the PB1-binding cavity (circled in orange). Other residues scatter mainly around the head domain of PAC. It is possible that compound **312** binds to PAC near these residues, resulting in the discrepancy between the inhibition of H5N1 RNP activities and the inhibition of other strains. Further investigation is needed to reveal the exact mechanism of action by compound **312**.

In summary, we have identified a hit compound (compound **221**) targeting influenza PAC domain by SPR screening. It can inhibit influenza RNP activities and impede viral replication. Twenty-three analogs of compound **221** were obtained by chemical synthesis or from purchase. Twelves of them could inhibit influenza RNP activity, among which compound **312** was identified as the most promising analog. Compound **312** could inhibit RNP, attenuate viral yield and bind directly to PAC at micromolar range.

## Methods

### Cell, virus and commercially available compound

293T and MDCK cells were routinely cultured in Minimum Essential Medium (MEM, Gibco) supplemented with 10% (v/V) fetal bovine serum (FBS, Gibco) at 37 °C in a humidified 5% CO_2_ incubator. Influenza virus A/WSN/33 and A/PR/8/34 were used for viral yield reduction assays. All commercially available compounds were purchased mainly from InterBioScreen (Moscow, Russia) and SPEC (Zoetermeer, Netherlands).

### Bioactivity assay

#### Protein expression and purification

Residues 256–716 of A/WSN/33 PAC was cloned into vector pET28a and expressed in *Escherichia coli* BL21(DE3) pLysS for 16 hrs at 16 °C. Cell pellet was resuspended with 20 mM Tris, 200 mM NaCl, 1% Glycerol, 1 mM TCEP, pH 8.0. The suspension was then lysed by sonication, and the lysate was centrifuged at 16,000 g for 1 hr at 4 °C. The supernatant was passed through a HisTrap column (GE Healthcare) for purification. The bound protein was eluted with 20 mM sodium phosphate, 200 mM NaCl and 500 mM imidazole. The eluate was then loaded to Superdex 200 (GE Healthcare) in 20 mM Tris, 200 mM NaCl, 1% Glycerol, pH 8.0 for further purification. The protein was purified to >95% purity, as assessed by polyacrylamide gel electrophoresis.

### Surface plasmon resonance (SPR) for screening and kinetic study

Purified PAC was diluted to 50 μg/ml by immobilization buffer (Phosphate buffer saline, pH 7.4) and was immobilized onto CM5 sensor chips with their primary amide groups using Amine Coupling Kit (Pharmacia). The CM5 sensor chip was activated by injecting 100 μl of 1:1 of EDC (0.4 μM 1-ethyl-3-(3-dimethylaminopropyl)-carbodiimide in water) and NHS (0.1 μM N-hydroxysuccinimide in water) at 5 μl/min. 50 μg/ml PAC was then injected into the flow cell at 5 μl/min. 50 μl of 1 M ethanolamine (pH 8.5) was then injected at 5 μl/min to inactivate the excess reactive group on the sensor surface. A control uncoupled sensor surface was also treated similarly without the addition of protein. The chip was then equilibrated with running buffer (Phosphate buffer saline, 5% DMSO, pH 7.4) before measurements. In both screening study and kinetic study, the flow rate was fixed at 40 μl/min and temperature was fixed at 25 °C.

For screening study, 4781 RU of purified PAC is immobilized to the CM5 chip. Compounds from DMSO stock were prepared and diluted in running buffer. Depending on the solubility of chemical, final concentration of chemical spanned from 25 to 100 μM. Using the “binding analysis” program, samples were injected into both coupled and uncoupled flow cells for 1 min. Binding surface was regenerated by the injection of 2 pulses of regeneration buffer (25 mM NaOH, 50 mM NaCl) onto chip surfaces. In addition, solvent control was injected for double referencing. DMSO calibration was implemented to correct for solvent effect.

For kinetic measurements, 3383 RU of purified PAC is immobilized to the CM5 chip. Compound 312 was serially diluted in running buffer and injected into both coupled and uncoupled flow cells for 2 mins. Binding surface was regenerated by regeneration buffer (25 mM NaOH, 50 mM NaCl). The association constant, dissociation constant and the affinity of the interaction are calculated by BIAevaluation v. 4.1 using the ‘1:1 Langmuir binding’ model.

### Cytotoxicity of the selected compounds

Cytotoxicity of compounds were assessed by 3-(4,5-dimethylthiazol-2-yl)-2,5-diphenyltetrazolium bromide (MTT) assay. Cells were seeded on a 96-well microtiter plate in MEM supplemented with 10% FBS. After overnight culture, cells were treated with the compounds. On the day of harvest, MTT (USB Corporation) in phosphate buffered saline was freshly prepared. MTT solution was added to each well and the plates were incubated at 37 °C for 2 hrs. DMSO was then added to dissolve the formazan. Absorbance at 540 nm was measured by VICTOR 3 Multilabel plate reader (Perkin Elmer).

### RNP reconstitution assay

Plasmids pcDNA-PB1, pcDNA-PB2, pcDNA-PA, pcDNA-NP, pEGFP and pPolI-Luc-RT have been described previously^30,31^. 1 × 10^5^ 293T cells were seeded on a 96-well microtiter plate overnight. 125ng of pcDNA-PB1, pcDNA-PB2, pcDNA-PA, pcDNA-NP, pPolI-Luc-RT and pEGFP were co-transfected to 293T cells to reconstitute the RNP complexes with Lipofectamine 2000 (Invitrogen). After 6 hrs of transfection, growth medium with candidate compounds were added. 24 hrs later, the luciferase activity was assayed by Steady-Glo luciferase substrate (Promega). The fluorescence from GFP expression and luminescence were read with VICTOR 3 Multilabel plate reader (Perkin Elmer).

### Viral yield assay

MDCK cells were seeded into 24-well plate at 1 × 10^5^ cells/well in MEM medium, supplemented with 10% FBS, and incubated at 37 °C for 24 hrs. Cells were washed with PBS and infected with 100 µl influenza A/WSN/33 or A/PR/8/34 virus at MOI of 0.01. After incubating at 37 °C for 1 hr, the virus particles were removed and the cell monolayer was washed with PBS. Medium with various concentration of chemical compounds was then added to cells and incubated at 37 °C for 24 hrs. Viral titer was determined by standard plaque assay with MDCK cells. *In vitro* cell infection experiments with A/WSN/33 or A/PR/8/34 viruses were performed under biosafety level 2 (BSL-2) laboratory conditions.

### Microscale thermophoresis

Microscope Thermophoresis experiments were performed using 1:1 mixture of PAC and compounds in standard grade capillaries (Nanotemper Technologies). Samples were incubated at 25 °C within the capillaries for 5 mins prior to measurement. All measurements were conducted using Monolith NT.LabelFree instrument (NanoTemper Technologies) at 25 °C. Assays were conducted at 20% IR-laser power and Medium MST powers. The buffer used for the experiment was 20 mM Tris, 200 mM NaCl and 15% Glycerol, pH 8. Stock solutions of test compounds were first diluted in buffer to their respective initial concentrations. A two-fold dilution series of the compounds were then prepared for MST measurement.

### Statistical analysis

Errors bars are given as the standard deviation. Significance of differences were analyzed using two-tailed t-test. All analyzes were performed using Microsoft excel. Statistics with p value < 0.05 were considered as significant.

### Data availability

Procedures for chemical synthesis of compound 221 analogs and data generated or analysed in this study are included in this published article (and its Supplementary material).

### Chemicals

#### 2-[(2-(Piperazino)ethyl)amino]-3-phenylquinazolin-4(3H)-one (S1a)

A mixture of phenylisocyanate (0.01 mol) solution in toluene and 1.67 g (0.01 mol) 2- parathesin was refluxed for 5 hrs. After cooling to room temperature, the mixture was filtered and the residue was washed by toluene to afford product as a solid. A mixture of Ethyl 2-(3-phenylureido)benzoate (0.01 mol), ethanol (40 ml), and triethylamine (0.03 mol) was refluxed for 2 hrs. After cooling to room temperature, the mixture was filtered and the residue was washed by ethanol to afford product as a solid. A mixture of 3-Phenylquinazoline-2,4(1H,3H)-dione (0.001 mol) and phosphorus oxychloride (0.02 mol) was refluxed for 8 hrs. After cooling to ambient temperature, the solution was evaporated in vacuo. The residue was dissolved in dichloromethane (DCM) and purified by column chromatography on silica gel to get the product. A mixture of 2-chloro-3-phenylquinazolin-4(3H)-one (0.001 mol), 3- (morpholino)propanamine or 2-(piperazino)ethanamine (0.001 mol), triethylamine (0.003 mol) and n-butyl alcohol (3 ml) was refluxed for 6 hrs. After cooling to room temperature, the mixture was filtered and the residue was dried to afford 2-[(2-(piperazino)ethyl)amino]-3-phenylquinazolin-4(3H)-one **(S1a)**.

#### 2-[(3-Morpholinopropyl)amino]-3-(2-methylphenyl)quinazolin-4(3H)-one (S1b)

A mixture of 2-methylphenyl isocyanate (0.01 mol) solution in toluene and 1.67 g (0.01 mol) 2- parathesin was refluxed for 5 hrs. After cooling to room temperature, the mixture was filtered and the residue was washed by toluene to afford the product as a solid. A mixture of Ethyl 2-[3-(2-methylphenyl)ureido]benzoate (0.01 mol), ethanol (40 ml), and triethylamine (0.03 mol) was refluxed for 2 hrs. After cooling to room temperature, the mixture was filtered and the residue was washed by ethanol to afford the product as a solid. A mixture of 3-(2-methylphenyl)quinazoline-2,4(1H,3H)-dione (0.001 mol) and phosphorus oxychloride (0.02 mol) was refluxed for 8 hrs. After cooling to ambient temperature, the solution was evaporated in vacuo. The residue was dissolved in DCM and purified by column chromatography on silica gel to get the product. A mixture of 2- Chloro-3-(2-methylphenyl)quinazolin-4(3H)-one (0.001 mol), 3- (morpholino)propanamine or 2-(piperazino)ethanamine (0.001 mol), triethylamine (0.003 mol) and n-butyl alcohol (3 ml) was refluxed for 6 hrs. After cooling to room temperature, the mixture was filtered and the residue was dried to 2-[(3-morpholinopropyl)amino]-3-(2-methylphenyl)quinazolin-4(3H)-one **(S1b)**.

#### 2-[(3-Morpholinopropyl)amino]-3-(4-methylphenyl)quinazolin-4(3H)-one (S1c)

A mixture of 4-methylphenyl isocyanate (0.01 mol) solution in toluene and 1.67 g (0.01 mol) 2-parathesin was refluxed for 5 hrs. After cooling to room temperature, the mixture was filtered and the residue was washed by toluene to afford the product as a solid. A mixture of Ethyl 2-[3-(4-methylphenyl)ureido]benzoate (0.01 mol), ethanol (40 ml), and triethylamine (0.03 mol) was refluxed for 2 hrs. After cooling to room temperature, the mixture was filtered and the residue was washed by ethanol to afford the product as a solid. A mixture of 3-(4-methylphenyl)quinazoline-2,4(1H,3H)-dione (0.001 mol) and phosphorus oxychloride (0.02 mol) was refluxed for 8 hrs. After cooling to ambient temperature, the solution was evaporated in vacuo. The residue was dissolved in DCM and purified by column chromatography on silica gel to get the product. A mixture of 2-Chloro-3-(4-methylphenyl)quinazolin-4(3H)-one (0.001 mol), 3- (morpholino)propanamine or 2-(piperazino)ethanamine (0.001 mol), triethylamine (0.003 mol) and n-butyl alcohol (3 ml) was refluxed for 6 hrs. After cooling to room temperature, the mixture was filtered and the residue was dried to afford 2-[(3-morpholinopropyl)amino]-3-(4-methylphenyl)quinazolin-4(3H)-one (**S1c**).

#### 3-(4-Fluorophenyl)-2-((3-morpholinopropyl)amino)quinazolin-4(3H)-one (S1d)

A mixture of 4-fluorophenyl isocyanate (0.01 mol) solution in toluene and 1.67 g (0.01 mol) 2-parathesin was refluxed for 5 hrs. After cooling to room temperature, the mixture was filtered and the residue was washed by toluene to afford the product as a solid. A mixture of Ethyl 2-[3-(4-fluorophenyl)ureido]benzoate (0.01 mol), ethanol (40 ml), and triethylamine (0.03 mol) was refluxed for 2 hrs. After cooling to room temperature, the mixture was filtered and the residue was washed by ethanol to afford the product as a solid. A mixture of 3-(4-fluorophenyl)quinazoline- 2,4(1H,3H)-dione (0.001 mol) and phosphorus oxychloride (0.02 mol) was refluxed for 8 hrs. After cooling to ambient temperature, the solution was evaporated in vacuo. The residue was dissolved in DCM and purified by column chromatography on silica gel to get the product. A mixture 2-Chloro-3-(4-fluorophenyl)quinazolin-4(3H)-one (0.001 mol), 3- (morpholino)propanamine or 2-(piperazino)ethanamine (0.001 mol), triethylamine (0.003 mol) and n-butyl alcohol (3 ml) was refluxed for 6 hrs. After cooling to room temperature, the mixture was filtered and the residue was dried to afford 3-(4-Fluorophenyl)-2-((3-morpholinopropyl)amino)quinazolin-4(3H)-one **(S1d)**.

#### Diethyl 2-(1-phenylethyl)malonate (1)

To a solution of diethyl malonate (1 eq) in dimethylformamide (DMF) in with ice bath, NaH(1.2 eq) was added and stirring was continued for 30 mins at 0 °C. (1-bromoethyl)benzene (1 eq) was then added. The reaction mixture was stirred for 5 hrs at room temperature. The reaction was then quenched with NH_4_Cl aq. extraction with ethyl acetate (EA), crude product was obtained, and was purified by silica gel column chromatography to afford compound **(1)**.

#### 1-bromo-2-(methoxymethoxy)ethane (2)

To a solution of 2-bromoethanol (1 eq) in DCM with ice bath, N,N-diisopropylethylamine (2 eq) was added. Methoxymethyl chloride (1.5 eq) was added slowly and stirred for 4 hrs at room temperature. The reaction was quenched with a saturated aqueous NaHCO_3_ solution and extracted with DCM. Crude product was purified by silica gel column chromatography to afford compound **(2)**.

#### 1,3-diphenylpyrimidine-2,4,6(1H,3H,5H)-trione (3)

To a solution of 1,3-diphenylurea(1 eq) in EtOH, NaOEt (1.3 eq) was added. Diethyl malonate (1.2 eq) was then added and stirred for 1 hr at room temperature and then reflux overnight. The reaction was quenched with 1M HCl at 0 °C, extracted with EA. Crude product was purified by silica gel column chromatography to afford compound **(3)**.

#### 6-chloro-1,3-diphenylpyrimidine-2,4(1H,3H)-dione (4)

Compound **(3)** was added to POCl_3_ (10 eq) and water (1.1 eq) at room temperature and stirred for 50 min, and then stirred for 5 hrs at 110 °C. Reaction mixture was cooled to room temperature, ice water and saturated aqueous NaHCO_3_ solution was added, and extracted with EA. Crude product was purified by silica gel column chromatography to afford compound **(4)**.

#### 6-azido-1,3-diphenylpyrimidine-2,4(1H,3H)-dione (5)

To a solution of compound **(4)** (1 eq) in DMF, 1.25 eq NaN_3_ was added and stirred overnight at room temperature. The reaction was then quenched with water, and extracted with EA. Crude product was purified by silica gel column chromatography to afford compound (5).

#### 6-amino-1,3-diphenylpyrimidine-2,4(1H,3H)-dione (6)

To a solution of compound **(5)** in MeOH, Palladium on carbon (Pd/C) (10%) was added, it was then hydrogenated for 5 hrs. Then Pd/C was filtered from the solution, the filtrate was concentrated to obtain the product **(6)**.

#### 6-(methylamino)-1,3-diphenylpyrimidine-2,4(1H,3H)-dione (7)

To a solution of compound **(4)** (1 eq) in EtOH, MeNH_2_/MeOH (1.5 eq) was added, the reaction mixture was stirred at 80 °C overnight. The reaction was concentrated to obtain the product **(7)**.

#### 6-benzyl-5-hydroxy-1,3-diphenylpyrido[2,3-d]pyrimidine- 2,4,7(1H,3H,8H)-trione (8)

To a solution of compound **(6)** (1 eq) in PhOPh, diethyl 2-benzylmalonate (1 eq) was added. The mixture was heated to 220–250 °C, stirred overnight, and cooled to room temperature. The reaction was then quenched with water, and extracted with EA. Crude product was purified by silica gel column chromatography to afford compound **(8)**.

#### 6-benzyl-5-hydroxy-8-(2-(methoxymethoxy)ethyl)-1,3-diphenylpyrido[2,3- d]pyrimidine-2,4,7(1H,3H,8H)-trione (9)

To a solution of compound **(8)** (1 eq) in DMF, K_2_CO_3_ (1.5 eq) was added, stirred for 30 min. 1-bromo-2-(methoxymethoxy)ethane (1.3 eq) was added, and the reaction was stirred for 4 hrs. The reaction was then quenched with water, and extracted with EA, Crude product was purified by silica gel column chromatography to afford compound **(9)**.

#### 5-hydroxy-8-methyl-1,3-diphenylpyrido[2,3-d]pyrimidine- 2,4,7(1H,3H,8H)-trione (10)

To a solution of compound **(7)** (1 eq) in PhOPh, diethyl malonate (1 eq) was added, then the mixture was heated to 220–250 °C. It was stirred overnight, and cooled to room temperature. The reaction was then quenched with water, and extracted with EA. Crude product was purified by silica gel column chromatography to afford compound **(10)**.

#### 6-benzyl-5-hydroxy-8-(2-hydroxyethyl)-1,3-diphenylpyrido[2,3- d]pyrimidine-2,4,7(1H,3H,8H)-trione (S2a)

To a solution of compound **(9)** in MeOH, HCl/MeOH was added and stirred for 4 hrs. The reaction was concentrated to obtain the product **(S2a)**.

#### 5-hydroxy-1,3-diphenyl-6-(1-phenylethyl)pyrido[2,3-d]pyrimidine- 2,4,7(1H,3H,8H)-trione (S2b)

To a solution of compound **(6)** (1 eq) in PhOPh, compound (1) (1 eq) was added, then the mixture was heated to 220–250 °C, stirred overnight, and cooled to room temperature. The reaction was then quenched with water, and extracted with EA. Crude product was purified by silica gel column chromatography to afford compound **(S2b)**.

#### 5-hydroxy-8-methyl-1,3-diphenyl-6-(1-phenylethyl)pyrido[2,3- d]pyrimidine-2,4,7(1H,3H,8H)-trione (S2c)

To a solution of compound **(7)** (1 eq) in PhOPh, compound (1) (1 eq) was added. The mixture was heated to 220–250 °C, stirred overnight, and cooled to room temperature. The reaction was then quenched with water, and extracted with EA. The crude product was purified by silica gel column chromatography to afford compound **(S2c)**.

#### 5-methoxy-8-methyl-1,3-diphenyl-6-(1-phenylethyl)pyrido[2,3- d]pyrimidine-2,4,7(1H,3H,8H)-trione (S2d)

To a solution of compound **(S2c)** in DMF, K_2_CO_3_ (1.5 eq) was added and stirred for 30 min. MeI(1.3 eq) was then added, and the reaction was stirred for 4 hrs. The reaction was then quenched with water, and extracted with EA. Crude product was purified by silica gel column chromatography to afford compound **(S2d)**.

#### 8-acetyl-5-hydroxy-1,3-diphenyl-6-(1-phenylethyl)pyrido[2,3- d]pyrimidine-2,4,7(1H,3H,8H)-trione (S2e)

To a solution of compound **(S2b)** (1 eq) in EtOH, acetic anhydride (1.05 eq) was added at room temperature. The solution was stirred for 15 min then concentrated in vacuo to give compound **(S2e)**.

#### 5-hydroxy-8-methyl-1,3-diphenyl-6-(phenylamino)pyrido[2,3- d]pyrimidine-2,4,7(1H,3H,8H)-trione (S2f)

To a solution of compound (10) in H_2_O under N_2_, PhI(OAc)_2_(1 eq) and Na_2_CO_3_(2 eq) was added. The reaction was stirred for 4 hrs at room temperature. The reaction was then quenched with water, and extracted with EA. Crude product was obtained.

The crude product was dissolved in toluene, aniline (1 eq) was added, the reaction mixture was refluxed overnight after evaporation of the solvent and was purified by silica gel column chromatography to afford compound **(S2f)**.

## Electronic supplementary material

Supplementary Information
