# Supplementary material for: Identification of influenza polymerase inhibitors targeting C-terminal domain of PA through surface plasmon resonance screening
Source: Sci Rep. 2018 Feb 2;8:2280. doi: 10.1038/s41598-018-20772-9 (PMC5797126; doi:10.1038/s41598-018-20772-9)
Supplement: Supplementary file 1 — Supplementary Information [file 41598_2018_20772_MOESM1_ESM.pdf]

Supplementary Information

Identification of influenza polymerase inhibitors targeting C-terminal domain of PA through surface plasmon resonance screening

Chun-Yeung Lo, Olive Tin-Wai Li,Wen-Ping Tang, Chun Hu, Guo Xin Wang, Jacky Chi-Ki Ngo, David Chi-Cheong Wan, Leo Lit-Man Poon, Pang-Chui Shaw*

* Corresponding Author

Supplementary Figure

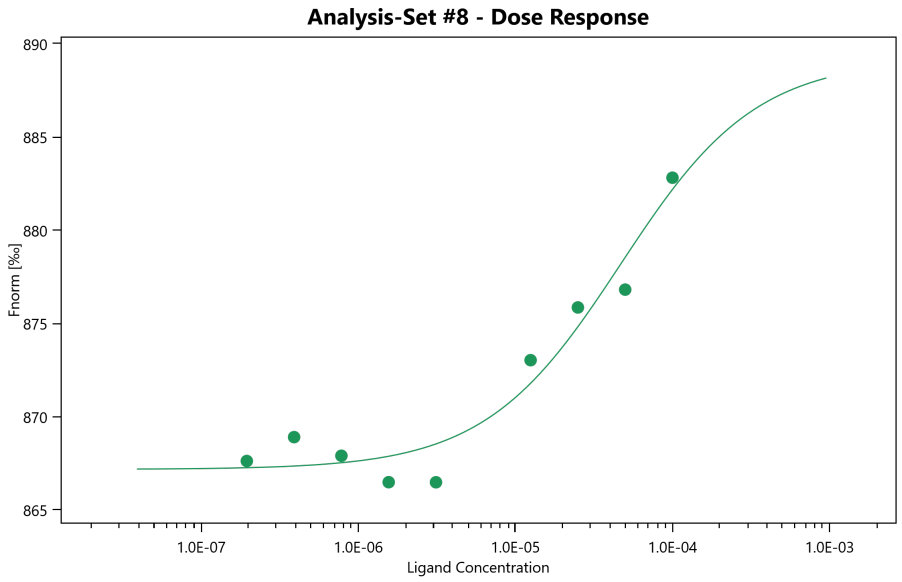

Figure S1. MST study of compound **387** with PAC.

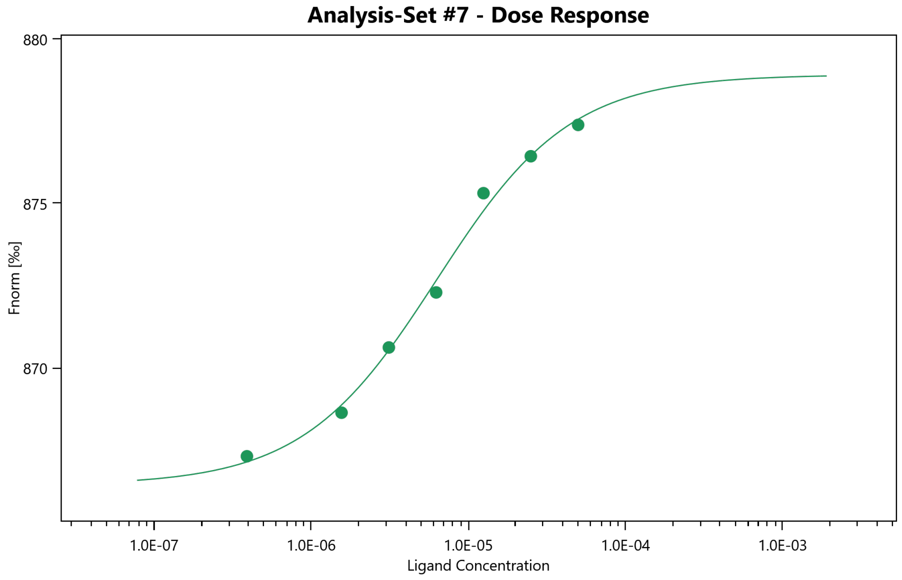

Figure S2. MST study of compound **392** with PAC.

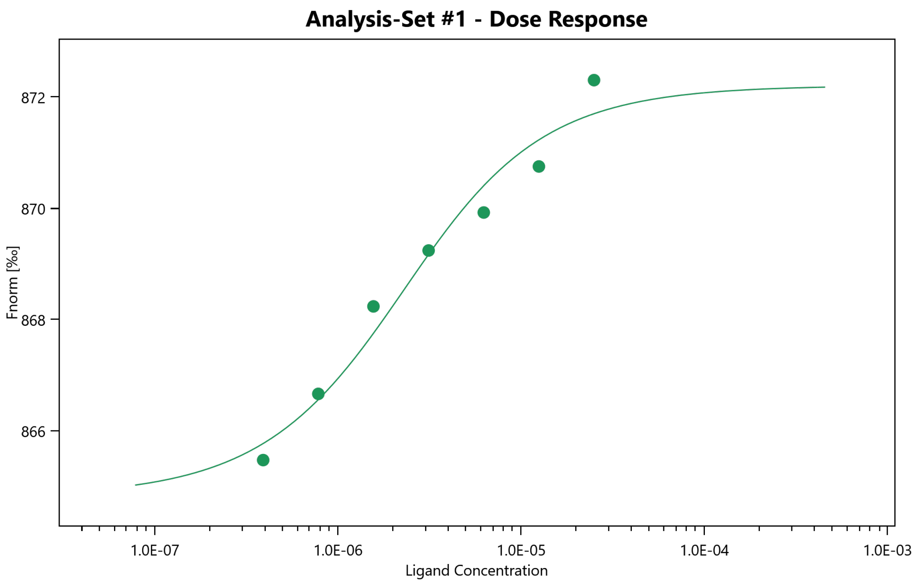

Figure S3. MST study of compound **S2a** with PAC.

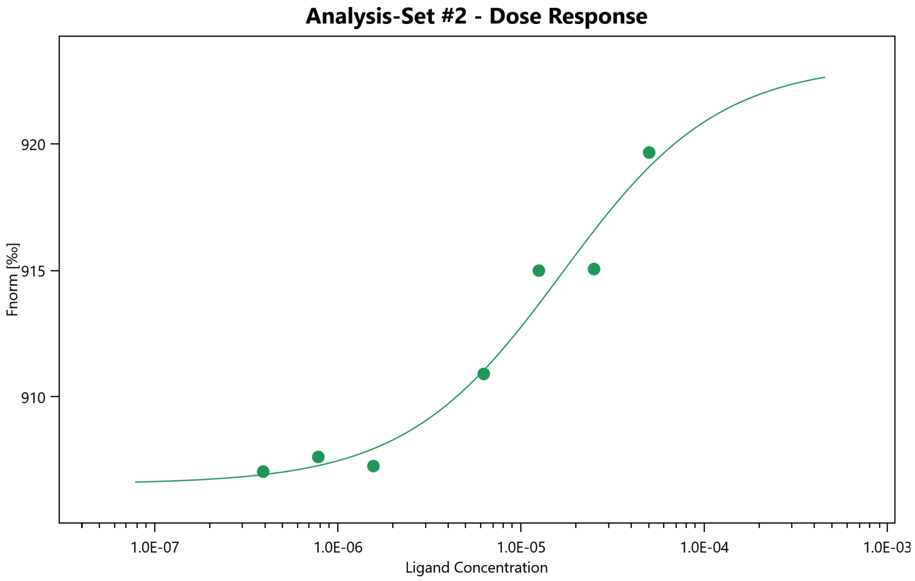

Figure S4. MST study of compound **S2b** with PAC.

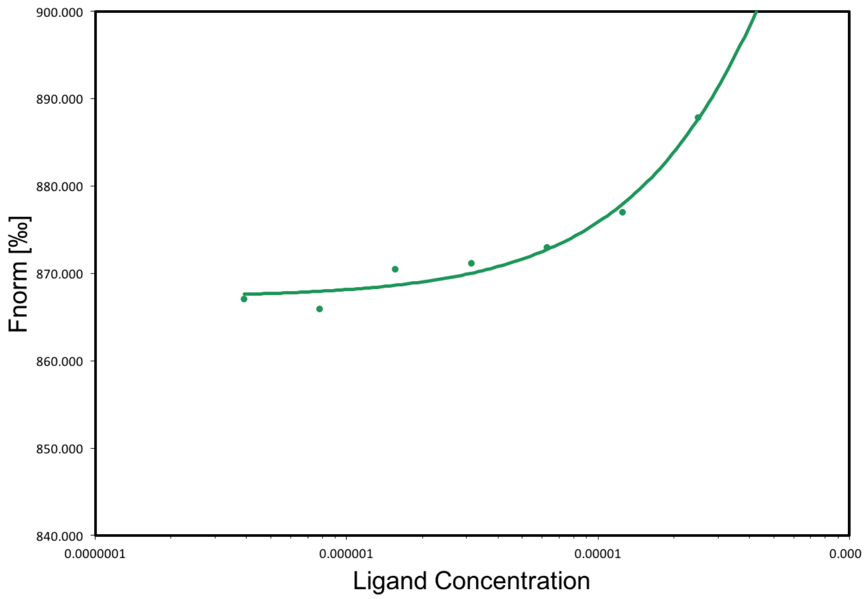

Figure S5. MST study of compound **S2d** with PAC.

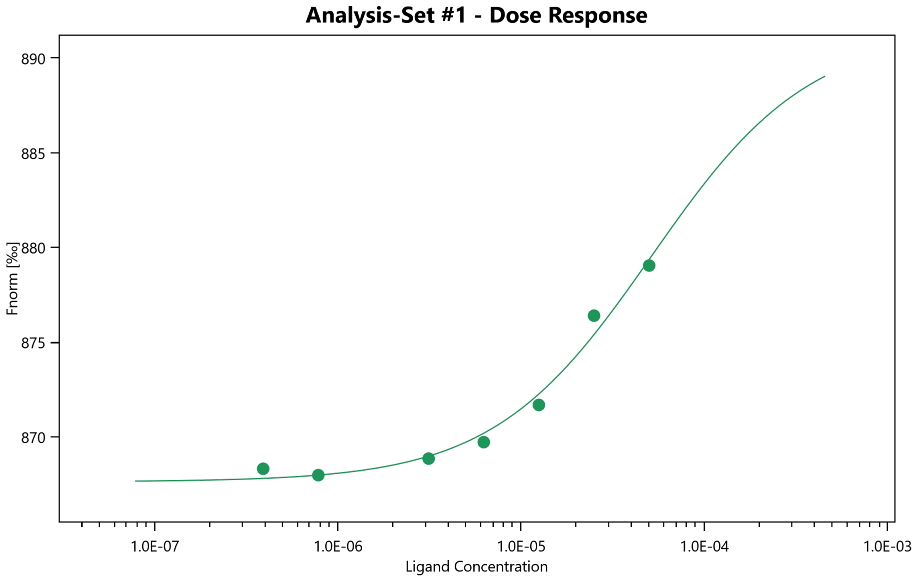

Figure S6. MST study of compound **S2e** with PAC.

```
H5N1 257                  IEPFLKTTPRPLRLPDGPPCSQRSKFLLMDALKLSIEDPSHEGE
H1N1                      IEPFLKSTPRPLRLPDGPPCSQRSKFLLMDALKLSIEDPSHEGE
H2N2                      IEPFLKTTPRPIRLPDGPPCSQRSKFLLMDALKLSIEDPSHEGE
H3N2                      IEPFLKTTPRPIRLPDGPPCFQRSKFLLMDALKLSIEDPSHEGE
                          ******:****:******** ***********************

H5N1 301  GIPLYDAIKCMKTFFGWREPNIIKPHEKGINPNYLLAWKQVLAELQDIENEDKIPKTKNM
H1N1      GIPLYDAIKCMRTFFGWKEPNVVKPHEKGINPNYLLSWKQVLAELQDIENEEKIPRTKNM
H2N2      GIPLYDAIKCMRTFFGWKEPYVVKPHEKGINPNYLLSWKQVLAELQDIENEEKIPRTKNM
H3N2      GIPLYDAIKCMRTFFGWKEPYIVKPHEKGINPNYLLSWKQVLAELQDIENEEKIPRTKNM
          ***********:*****:** ::*************:**************:***:****

H5N1 361  KKTSQLMWALGENMAPEKVDFEDCKDIDDLKQYHSDEPELRSLASWIQNEFNKACELTDS
H1N1      KKTSQLKWALGENMAPEKVDFDDCKDVGDLKQYDSDEPELRSLASWIQNEFNKACELTDS
H2N2      KKTSQLKWALGENMAPEKVDFDDCRDTSDLKQYDSDEPELRSLSSWIQNEFNKACELTDS
H3N2      KKTSQLKWALGENMAPEKVDFDNCRDVSDLKQYDSDEPELRSLSSWIQNEFNKACELTDS
          ****** **************::*:* .*****.*********:****************

H5N1 421  SWIELDEIGEDVAPIEHIASMRRNYFTAEVSHCRATEYIMKGVYINTALLNASCAAMDDF
H1N1      SWIELDEIGEDAAPIEHIASMRRNYFTAEVSHCRATEYIMKGVYINTALLNASCAAMDDF
H2N2      IWIELDEIGEDVAPIEHIASMRRNYFTAEVSHCRATEYIMKGVYINTALLNASCAAMDDF
H3N2      TWIELDEIGEDVAPIEYIASMRRNYFTAEVSHCRATEYIMKGVYINTALLNASCAAMDDF
           **********.****:*******************************************

H5N1 481  QLIPMISKCRTKEGRRRTNLYGFIVKGRSHLRNDTDVVNFVSMEFSLTDPRLEPHKWEKY
H1N1      QLIPMISKCRTKEGRRKTNLYGFIIKGRSHLRNDTDVVNFVSMEFSLTDPRLEPHKWEKY
H2N2      QLIPMISKCRTKEGRRKTNLYGFIIKGRSHLRNDTDVVNFVSMEFSLTDPRLEPHKWEKY
H3N2      QLIPMISKCRTKEGRRKTNLYGFIIKGRSHLRNDTDVVNFVSMEFSLTDPRLEPHKWEKY
          ****************:*******:***********************************

H5N1 541  CVLEIGEMLLRTAIGQVSRPMFLYVRTNGTSKIKMKWGMEMRRCLLQSLQQIESMIEAES
H1N1      CVLEVGDMLLRSAIGHVSRPMFLYVRTNGTSKIKMKWGMEMRRCLLQSLQQIESMIEAES
H2N2      CVLEIGDMLLRSAIGQVSRPMFLYVRTNGTSKIKMKWGMEMRRCLLQSLQQIESMIEAES
H3N2      CVLEIGDMLLRSAIGQMSRPMFLYVRTNGTSKIKMKWGMEMRRCLLQSLQQIESMIEAES
          ****:*:****:***::*******************************************

H5N1 601  SIKEKDMTKEFFENRSETWPIGESPKGVEEGSIGKVCRTLLAKSVFNSLYSSPQLEGFSA
H1N1      SVKEKDMTKEFFENKSETWPVGESPKGVEEGSIGKVCRTLLAKSVFNSLYASPQLEGFSA
H2N2      SVKEKDMTKEFFENKSETWPIGESPKGVEEGSIGKVCRTLLAKSVFNSLYASPQLEGFSA
H3N2      SVKEKDMTKEFFENKSETWPIGESPKGVEDGSIGKVCRTLLAKSVFNSLYASPQLEGFSA
          *:************:*****:*********:*******************:*********

H5N1 661  ESRKLLLIVQALRDNLEPGTFDLEGLYGAIEECLINDPWVLLNASWFNSFLTHALR
H1N1      ESRKLLLIVQALRDNLEPGTFDLGGLYEAIEECLINDPWVLLNASWFNSFLTHALR
H2N2      ESRKLLLVVQALRDNLEPGTFDLGGLYEAIEECLINDPWVLLNASWFNSFLTHALR
H3N2      ESRKLLLVVQALRDNLEPGTFDLEGLYEAIEECLINDPWVLLNASWFNSFLTHALR
          *******:*************** *** ****************************
```

Figure S7. Sequence alignment of A/WSN/33 (H1N1), A/Japan/305/1957 (H2N2), A/HK/1/68 (H3N2) and A/HK/156/97 (H5N1) PAC. Distinct residues of H5N1 PAC are highlighted in red.

Supplementary Table 1. Chemical structure of hit compounds from SPR screening.

Compound ID.	Chemical Structure	Compound ID.	Chemical Structure
57	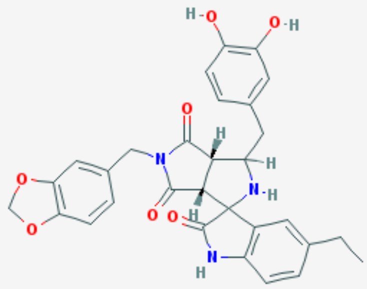	265	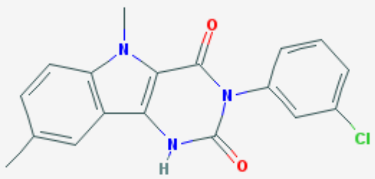
95	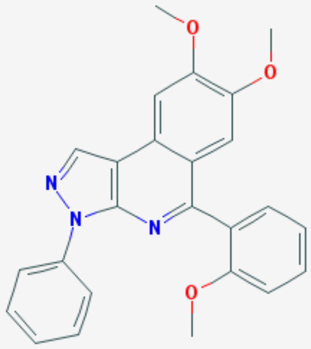	270	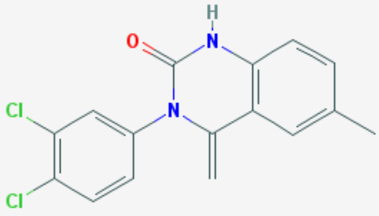
123	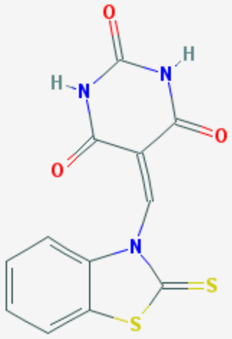	271	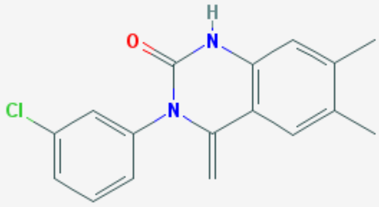

131	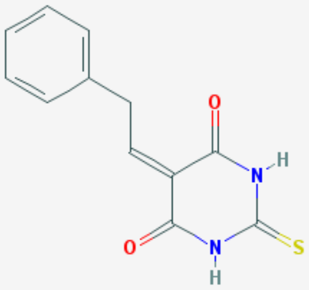	272	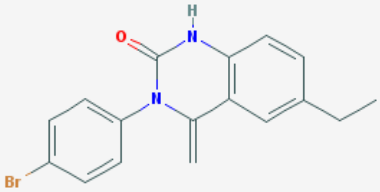
190	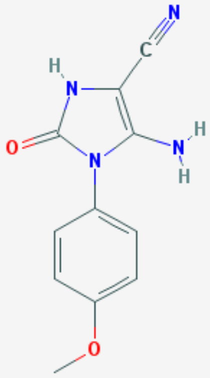	273	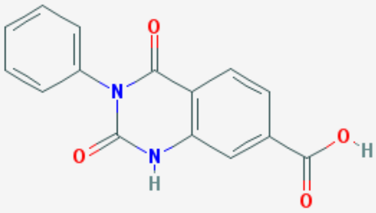
198	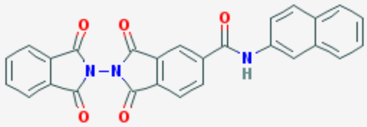	280	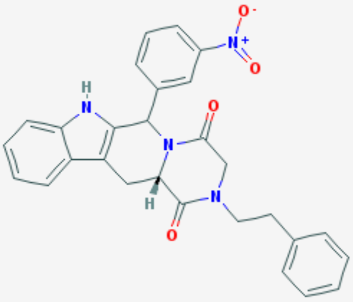
203	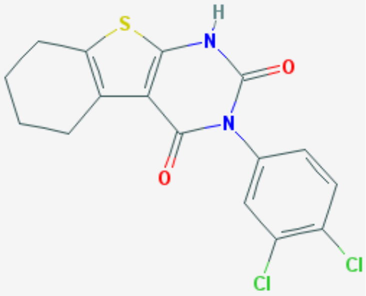	283	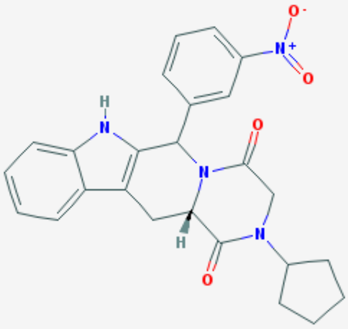

221

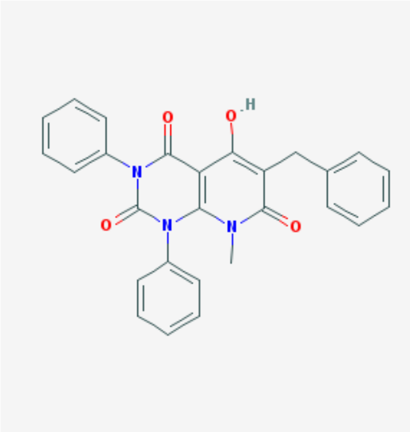

345

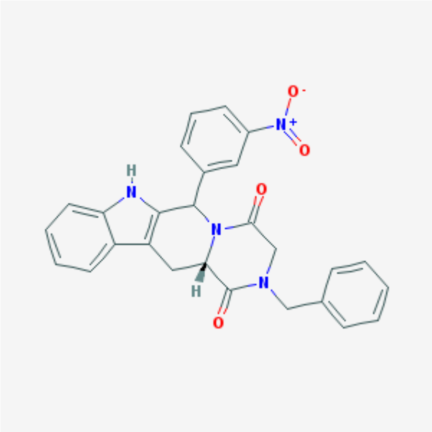

263

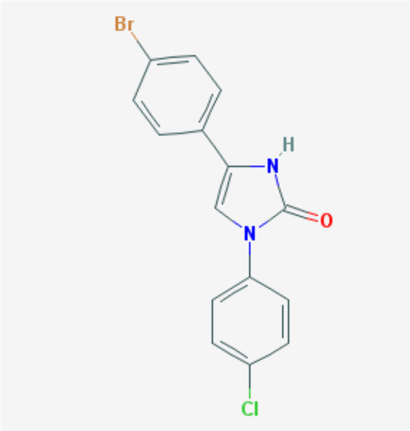

Supplementary Table2. Cytotoxicity of compounds

		CC_{50}[a] (μM)	
	Compound	293Ta	MDCK
Hit compounds from SPR screening	57	>50	N.D.
	95	>25	N.D.
	123	>50	N.D.
	131	>50	N.D.
	190	>100	N.D.
	198	>5	N.D.
	203	>100	N.D.
	221	>100	>100
	263	>100	N.D.
	265	>100	N.D.
	270	82.0 ± 2.5	N.D.
	271	>100	N.D.
	272	>100	N.D.
	273	>100	N.D.
	280	>100	N.D.
	283	>10	>10
	345	>100	N.D.
Analogues obtained through chemical modification	S1a	>100	N.D.
	S1b	>100	>100
	S1c	>100	N.D.
	S1d	>100	N.D.
	S2a	84.6 ± 17.30	>100
	S2b	>100	42.8 ± 9.0
	S2c	97.1 ± 16.10	N.D.
	S2d	66.6 ± 6.09	35.6 ± 9.24
	S2e	>100	53.1 ± 9.25
	S2f	>100	>100
Commercially available analogs	310	>100	>100
	312	>100	>100
	384	>100	>100
	385	>100	N.D.
	387	>100	>100
	389	>100	>100
	390	>100	N.D.
	391	>100	N.D.
	392	62.66 ± 18.7	87.03 ± 7.22
	394	>100	N.D.
	395	>100	N.D.
	396	>100	N.D.
	397	>100	N.D.

[a]293T or MDCK cells were incubated with test compounds for 24 hrs; CC50 is the concentration of test compounds which produces 50% cytotoxicity as determined by MTT assays; N.D., not determined; reported values represent means ± standard deviation of data from three independent experiments.
